# Prognostic value of circulating tumor cells and disseminated tumor cells in patients with ovarian cancer: a systematic review and meta-analysis

**DOI:** 10.1186/s13048-015-0168-9

**Published:** 2015-06-16

**Authors:** Long Cui, Joseph Kwong, Chi Chiu Wang

**Affiliations:** Department of Obstetrics and Gynecology, Prince of Wales Hospital, The Chinese University of Hongkong, Shatin, Hong Kong China

**Keywords:** Ovarian cancer, Circulating tumor cells, Disseminated tumor cells, Prognosis

## Abstract

Recent studies have shown diagnostic and prognostic values of circulating tumor cells (CTCs) and disseminated tumor cells (DTCs) in various cancers, including ovarian cancer. We aimed to evaluate the association of CTCs and/or DTCs with the clinical outcomes of ovarian cancer. Clinical studies of CTCs/DTCs of ovarian cancer were included for systematic review and meta-analysis. A total of 236 studies were screened but only 16 qualified studies with 1623 subjects were included. Odds ratio (OR) showed CTCs/DTCs were not significantly associated with serous carcinoma (OR = 0.71 [0.49, 1.05]), lymph node metastasis (OR 1.14 [0.67, 1.93]), and residual disease (OR 1.45 [0.90, 2.34]); but significantly associated with advanced tumor staging (OR = 1.90 [1.02, 3.56]). The overall pooled hazard ratio (HR) of CTCs/DTCs on OS and PFS/DFS was 1.94 [1.56– 2.40] and 1.99 [1.59–2.50], respectively. Subgroup analyses revealed that CTCs were significantly associated OS (HR 1.97 [1.50-2.58]) and PFS/DFS (HR 2.52 [1.83-3.48]), while DTCs was significantly associated OS (HR 1.89 [1.33, 2.68]) and PFS/DFS (HR 1.60 [1.17, 2.19]). Meta-analysis showed strong relationship of CTCs/DTCs with advanced staging, treatment response and poor prognosis in patients with ovarian cancer.

**Electronic supplementary material:**

The online version of this article (doi:10.1186/s13048-015-0168-9) contains supplementary material, which is available to authorized users.

## Introduction

Ovarian cancer is the most frequent cause of death amongst gynecological cancers worldwide. Majority of cases diagnosed in late stage of the disease and resulted in poor survival [1]. The five-year survival rate of patients with ovarian cancer is only around 30 % in Stage III or IV [2]. The reasons of delayed diagnosis are partly due to lack of sensitive signs and symptoms and effective screening methods [3]. Although survival has been improved with the use of cyto-reduction surgery along with platinum- and/or taxane-based chemotherapy, nearly 80 % eventually relapse within 5 years [4]. Therefore, methods that help detection of ovarian cancer in early stage and monitoring of tumor progression have great potential to improve survival of the patients.

It was considered that ovarian cancer spreads primarily through direct dissemination in the abdominal cavity. While the presence of disseminated tumor cells (DTCs) in bone marrow of patients with ovarian cancer have been reported [5, 6]. However, bone marrow sampling is rather an invasive procedure, which is not widely accepted in the clincial management. In recent years, focus has been shifted to the detection of circulating tumor cells (CTCs) in peripheral blood. CTCs are tumor cells release from the primary tumor and then circulate through the bloodstream, resulting in spreading to different organs and subsequent outgrowth of the tumor cells in new microenvironment. These CTCs thereby have the potential to contribute to the development of local and systematic relapses [7]. Either DTCs or CTCs have potential to predict prognosis and to monitor treatment efficacy in cancer patients. Presence of CTCs has been reported in several solid tumors, including breast [8], colorectal [9], lung [10], kidney [11], esophageal [12], liver [13], prostate [14], and pancreatic cancers [15, 16]. Studies of CTCs/DTCs in ovarian cancer patients had been investigated, most of them demonstrated that CTC or DTC is associated with poor clinical outcome [17–22]. However, other studies failed to show the positive correlation [5, 23, 24] and even demonstrated negative association in terms of progression free survival/disease free survival (PFS/DFS) and overall survival (OS) [25, 26]. The prognosis value of CTCs/DTCs in ovarian cancer remains controversial. A recent systematic review of CTCs and DTCs in ovarian cancer concluded the association of CTCs and DTCs with adverse clincopathological characteristics and poor clinical outcomes [27], but no appropriate statistics and detailed analysis were provided.

The objective of this study was to conduct a meta-analysis of published clinical studies of CTCs/DTCs in ovarian cancer and to investigate the association of CTCs/DTCs with clinical outcomes.

## Methods

**Fig. 1:**
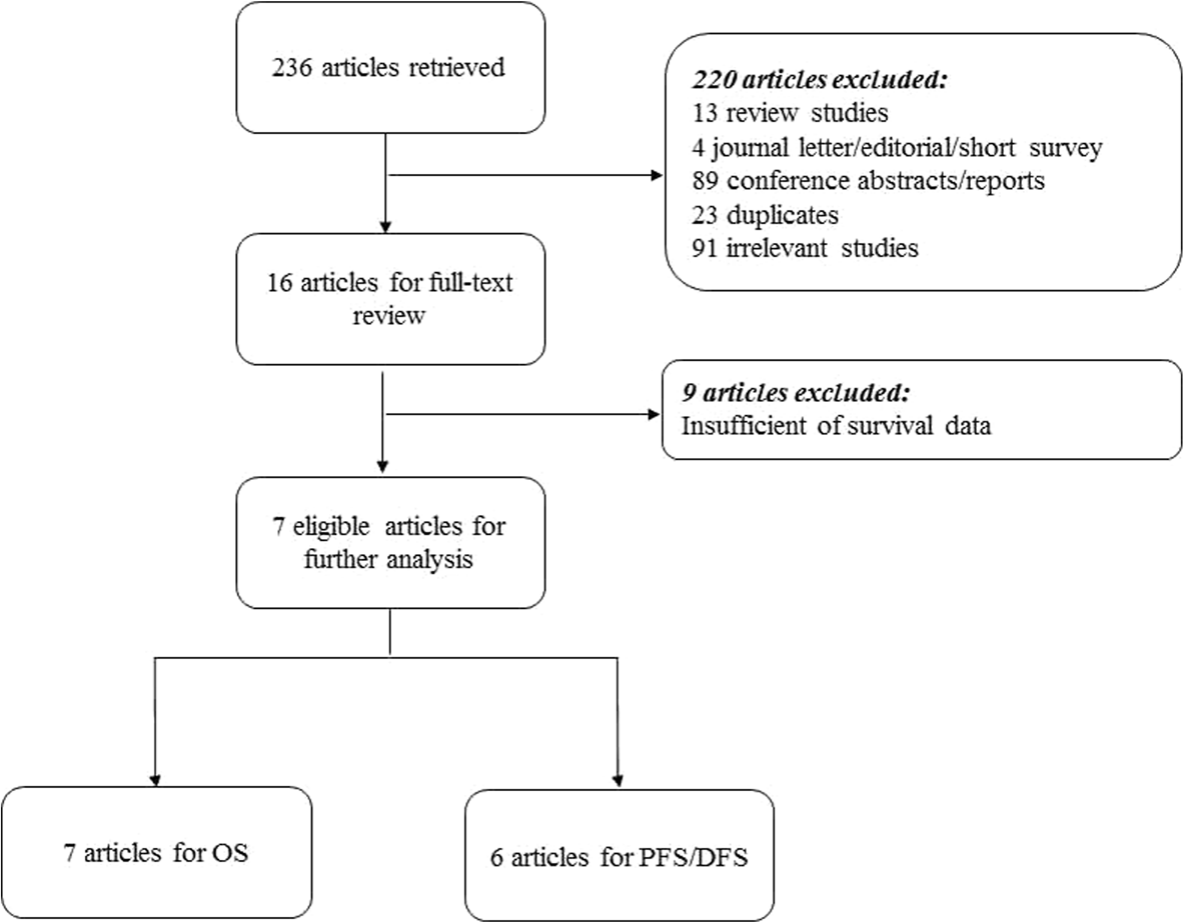
Flow diagram of study inclusion decisions

An independent systematic review of the literature across PubMed and EMBASE database was conducted on April 27, 2015. The search strategy included keywords such as “ovarian cancer”, “ovarian carcinoma”, “circulating tumor cell (s)”, “disseminated tumor cell (s)”, and “prognos*”. Only studies published in peer reviewed journals were included, data from letters and conference abstracts were not included. The study selection process is shown in Fig. 1 and search strategies and results are provided in Additional file 1.

We recorded the following information from each eligible study, including author’s name, publication year, number of participants, sampling time, methods and results of CTCs/DTCs detection, and OS and/or PFS/DFS. We also collected clinopathological parameters, including histology, lymph node metastasis, cancer stage, residual diseases and treatment response. Histological types of the ovarian cancer were classified mainly as serous carcinoma or non-serous carcinoma. Lymph node metastasis was confirmed by pathological examination in the lymph nodes collected during cyto-reduction surgery. According to FIGO staging of ovarian cancer, Stage I and Stage II were combined and Stage III and Stage IV were combined. Complete resection was referred to no residual disease for no residual tumor, minimal residual disease for residual tumor in 1 cm or less, and gross residual disease for residual tumor in greater than 1 cm [28, 29]. Treatment response was classified as platinum-sensitive as defined when the patients with platinum-free interval of ≥ 6 months or platinum-resistant disease as defined when the patients relapse ≥ 6 months from the end of first line platinum-base therapy [30].

Inclusion criteria included: 1) clinical studies measured CTCs/DTCs, regardless of randomized or case-controlled studies; prospective or retrospective studies; and detection methods; 2) study outcomes provided clinical and pathological information; and 3) studies provided information of survival outcomes such as OS and PFS/DFS. Studies were excluded based on: 1) laboratory studies without clinical outcomes; 2) review, editorials, and commentary articles; 3) no survival data or insufficient data to be extracted. Two reviewers independently screened titles and abstracts of the studies for inclusion and then retrieved the full text for details data extraction.

Meta-analyses were conducted according to the PRISMA and MOOSE Checklist and the quality of the included studies was assessed with the Newcastle-Ottawa Scale (NOS) for cohort studies [31]. (Additional file 2 and Additional file 3). We examined the association between CTCs/DTCs and clinopathological outcomes. Odds ratio (OR) was used as the measure of index to describe the association. Survival analysis for natural logarithm of HR (lnHR) and standard error (SE) were calculated. If these statistical variables were not explicitly provided in studies, lnHR, SE and p values will be calculated from the available numerical data and Kaplan-Meier survival curves according to Tierney et al. [32]. Fixed or random-effects models will be employed to calculate the pooled hazards ratio (HR) with 95 % confidence intervals (CIs) for survival. Heterogeneity among studies was conducted by Cochran-Mantel-Haenszel test with the Cochran’s Q test and P values. When P value less than 0.05, a random-effects model was used. Otherwise, a fixed-effects model was presented. Subgroup analyses of sample types and detection method were performed. Publication bias was assessed using funnel plot, then further examined by Begg and Egger’s test [33]. All statistical analyses were conducted using the software R/metafor version 2.14.0. P values less than 0.05 were considered statistically significant.

## Results

236 records were identified from the literature search. The selection processes are summarized in Fig. 1. After screening of titles and abstracts, 220 studies were excluded. 91 were irrelevant, 13 were review articles, 4 were letter/editorial/survey articles, 89 were conference abstracts/reports, and 23 were duplicated publications. Finally, 16 studies met the inclusion criteria for data extraction. We recorded the following information of each eligible study: author’s names, year of publication, number of patients analyzed, sampling timing, CTCs and/or DTCs studied, detection method of CTCs/DTCs, markers used for the detections, definition of positive CTCs/DTCs and survival data, and results of the studies. Details of the included studies are summarized in Table 1.

**Table 1:** Main characteristics of studies

Author	No. of Patients	Sampling time	CTCs or DTCs	Detection methods	Markers	Definition of positive	Outcome measures	Results
[17]	80	Pre-therapy	CTC	RT-PCR	MAGE-As	≥1 tumor-associated transcript over expressed	OS & PFS/DFS	Positive for PFS/DFS and OS
[18]	129	Pre-therapy	CTC	CAM-initiated CTC enrichment or identification	EpCAM, CD45 clone 5B1 and CD66b	iCTCs ≥ 5	OS & PFS/DFS	Positive for PFS/DFS and OS
[19]	143	Pre-therapy	CTC	RT-PCR	EpCAM, MUC1,or MUC16	≥1 tumor-associated transcript over expressed	OS & PFS/DFS	Positive for PFS/DFS and OS
[35]	216	Pre-therapy and post-therapy	CTC	RT-PCR	PPIC, GPX8, CDH3, TUSC3, COL3A1, LAMB1, MAM, ESRP2, AGR2, BAIAP2L1, TFF1, EpCAM	≥1 tumor-associated transcript over expressed	OS & PFS/DFS	Negative pre-therapy but positive for post-therapy
[34]	216	Pre-therapy	CTC	CellSearch (IHC)	EpCAM	≥2 cell stained	OS & PFS/DFS	Positive for PFS/DFS; negative for OS
[26]	122	Pre-therapy and/or post-therapy	CTC	RT-PCR	EpCAM, MUC-1, HER2 A45-B/B3 (BM)	≥1 tumor-associated transcript over expresse ≥1 CK cell positive (BM)	OS & PFS/DFS	CTC: positive for OS but negative for PFS/DFS; DTC: negative for PFS/DFS and OS
[20]	90	Pre-therapy	DTC	IHC	A45-B/B3	≥1 cell positive	OS & PFS/DFS	Positive for PFS/DFS and OS
[37]	112	Pre-therapy	DTC	IHC	A45-B/B3	≥1 CK cell positive	OS & PFS/DFS	Positive for PFS/DFS but negative for OS
[21]	62	Pre-therapy and post-therapy	DTC	IHC (Epimet® kit)	A45-B/B3	≥1 CK cell positive	OS & PFS/DFS	Positive for PFS/DFS and OS
[22]	69	Pre-therapy	DTC	IHC	A45-B/B3	≥1 CK cell positive	PFS/DFS	Positive for PFS/DFS
[24]	90	Pre-therapy	CTC/DTC	IHC	MOC-31	Presence of ≥2 rosettes (≥5 beads bound to a cell)	OS & PFS/DFS	Negative for PFS/DFS and OS
[5]	59	Pre-therapy	CTC	IHC	CK8 and 18 TFS-2, CK7, CK20 EGFR	≥1 cell positive	OS & PFS/DFS	Negative for PFS/DFS and OS
[25]	66	Pre-therapy	CTC	Cell invasion assay	EpCAM	≥1 cell positive	OS & PFS/DFS	Positive for PFS/DFS; Negative for OS
[23]	43	Pre-therapy and intra-therapy	CTC	CellSearch (IHC)	EpCAM	≥1 cell positive	OS & PFS/DFS	Negative for PFS/DFS and OS
[6]	57	Pre-therapy and intra-therapy	DTC	IHC (Epimet® kit)	A45-B/B3 EpCAM	≥1 cell positive	PFS/DFS	Positive for PFS/DFS
[38]	69	Pre-therapy	DTC	IHC	A45-B/B3	≥1 CK cell positive	PFS/DFS	Negative for PFS/DFS

*CTCs* Circulating tumor cells; *DTCs* Disseminated tumor cells; *RT-PCR* Reverse transcription-polymerase chain reaction; *IHC* Immunocytochemistry; *MAGE-A* Melanoma-associated antigens A; *PPIC* Peptidylprolyl isomerase C (cyclophilin C); *GPX8* Glutathione peroxidase 8; *CDH3* Cadherin-3; *TUSC3* Tumor suppressor candidate 3; *COL3A1* Collagen, Type III, alpha 1; *LAMB1* Laminin subunit beta-1; *MAM* Mammaglobin A; *ESRP2* Epithelial splicing regulatory protein 2; *AGR2* Anterior gradient protein 2 homolog; *BAIAP2L1* Brain-specific angiogenesis inhibitor 1-associated protein 2-like protein 1; *TFF1* Trefoil factor 1; *EpCAM* Epithelial cell adhesion molecule; *MUC-1* Mucin 1; *MUC-16* Mucin 16; *HER2* Human growth factor receptor 2; *A45-B/B3* Pan-cytokeratin antibody (CK 8, 18, 19); *MOC-31* Epithelial glycoprotein 2 mouse monoclonal antibody; *EGFR* Epithelial growth factor receptor; *iCTC* Invasive circulating tumor cells; *CK* Cytokeratin; *CAM* Cell adhesion matrix; *BM* Bone marrow; *OS* Overall survival; *PFS/DFS* Progression-free survival/disease-free survival

In total, there were 1623 patients, and the sample size of each study was ranged from 43 to 216. Most studies were published between 2002 and 2014, 4 studies from US, 11 studies from Europe and 1 study from Asia. There were 6 studies including 459 patients recorded the prognostic values of DTCs detected in bone marrow and 10 studies including 1164 patients recorded the prognostic values of CTCs detected in peripheral blood. Seven out of 16 studies had positive results of CTC/DTC effects on survival. Four out of 16 had negative results, remaining 5 studies had controversial conclusions.

Associations of CTCs/DTCs with clinicopathological parameters were analyzed (Table 2). Six studies [5, 17, 25, 34–36] with defined pathological diagnosis of serous carcinoma or non-serous carcinoma were included to study the relationship between CTCs/DTCs and histological types of the ovarian cancer. The estimated pooled OR was 0.72 (95 % CI: 0.48–1.06; Z = −1.71; P = 0.088 fixed-effect), demonstrating that CTCs were not associated with the tumour histology. The heterogeneity among studies was not significant (Q = 5.24, p = 0.387). Three studies [17, 35, 37] assessing metastasis in lymph node or not were included to study the relationship between CTCs/DTCs and lymph node metastasis. Of the results showed that CTCs/DTCs were not significantly associated with lymph node metastasis in ovarian cancer patients (pooled OR = 1.14; 95 % CI: 0.67–1.93; Z = 0.481; P = 0.630 fixed-effect). The heterogeneity among studies was not significant (Q = 3.82, p = 0.148). In six studies [5, 17, 25, 35–37], there was significant association between CTC and advanced tumor stage (Stage III-IV, pooled OR = 1.90; 95 % CI: 1.02–3.56; Z = 2.02; P = 0.044 fixed-effect), indicating that CTCs/DTCs were significantly increased with the risk of disease progression in ovarian cancer. The heterogeneity among studies was not significant (Q = 10.84, p = 0.055). Three studies [17, 25, 35], were included to study the relationship between CTCs/DTCs and debulking surgery, CTCs were not significantly associated with the optimal or suboptimal surgery in ovarian cancer patients (pooled OR = 1.45; 95 % CI: 0.90–2.34; Z = 1.53; P = 0.126 fixed-effect). However, one study [35] showed that DTCs significant association with residual diseases (OR = 2.31, CI: 1.19-4.50). The heterogeneity among studies was not significant (Q = 3.71, p = 0.157). Two studies [34, 35] assessing platinum sensitive or resistant were included to study the relationship between CTCs and treatment response, the result showed that CTCs were significantly associated with treatment response in ovarian cancer patients (pooled OR = 0.55; 95 % CI: 0.34–0.90; Z = −2.37; P = 0.017 fixed-effect). The heterogeneity among studies was not significant (Q = 0.930, p = 1.0000).

**Table 2:** Association of CTCs/DTCs and clinicopathological datasets

	No. of Studies (sample size)	OR (95 % CI)	Model	*OR, p* value	P-H	Begg’s test, *p* value
Serous carcinoma vs. Non-serous carcinoma	6 (789)	0.71 [0.49,1.05]	FE	0.0878	0.3876	0.719
FIGO stage III-IV vs. FIGO stage I-II	6 (687)	1.90 [1.02, 3.56]	FE	0.0438	0.0546	0.1361
Lymph node metastasis vs. No lymph node metastasis	3 (404)	1.14 [0.67, 1.93]	FE	0.6304	0.1484	1.0000
Suboptimal debulking vs. optimal debulking	2 (141)	0.78 [0.32, 1.88]	FE	0.5751	0.3232	1.0000
Platinum sensitive vs. Platinum resistant	2 (508)	0.55 [0.34, 0.90]	FE	0.0178	0.9298	1.0000

*FE* Fixed-Effects; *P-H P* value –Heterogeneity

**Fig. 2:**
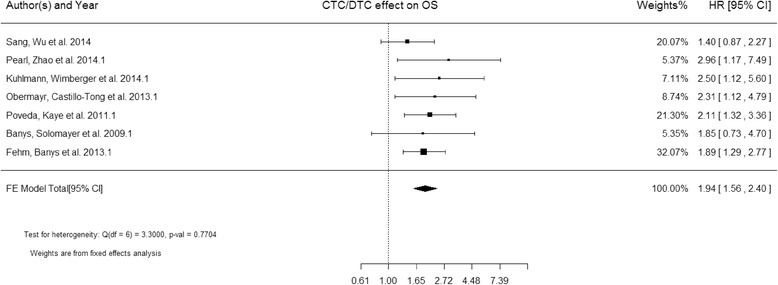
Forest plot of HRs for OS from 7 studies (965 patients)

OS was analyzed in 7 studies [17–19, 22, 34, 35, 37] including 965 patients in total. Since the heterogeneity across the studies was larger than 0.05 (Q = 3.3, P = 0.770), the estimated pooled HR for studies was calculated using a fixed effect model. The pooled HR showed that CTCs/DTCs were significantly associated with OS (HR = 1.94; 95 % CI: 1.56– 2.40; Z = 6.02; P < 0.0001 fixed effects), indicating CTCs/DTCs significantly increased the risk of overall mortality in ovarian cancer (Fig. 2).

**Fig. 3:**
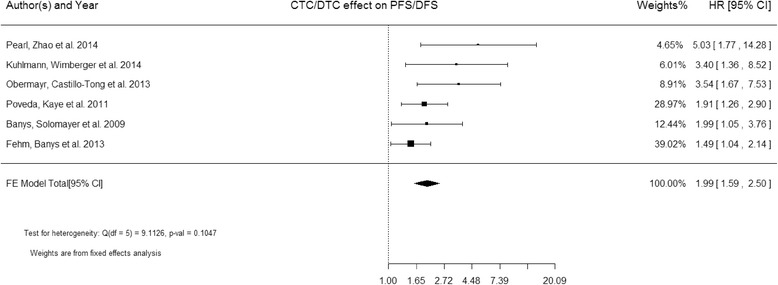
Forest plot of HRs for PFS/DFS from 6 studies (885 patients)

PFS/DFS were analyzed in 6 studies [18, 19, 22, 34, 35, 37] including 885 patients in total. Because the heterogeneity across the studies was also larger than 0.05 (Q = 9.11, P = 0.105), the estimated pooled HR for studies was calculated using a fixed effect model. The estimated pooled HR showed that CTCs/DTCs was also significantly associated with PFS/DFS (HR = 1.99; 95 % CI: 1.59–2.50; Z = 6.01; P < 0.0001 fixed effects), indicating CTCs/DTCs significantly increased with the risk of low survival in ovarian cancers (Fig. 3).

Results for subgroup analysis is summarized in Table 3. CTCs and DTCs could be detected in peripheral blood (PB) and bone marrow (BM), respectively, we divided the studies into either CTCs or DTCs subgroups to investigate the influence of sampling types on the survival of patients with ovarian cancer. CTCs from PB were detected and correlated with PFS/DFS and OS in 4 studies [18, 19, 34, 35] and 5 studies [17–19, 34, 35], respectively. The results showed that CTCs were significantly associated with both PFS/DFS (HR =2.52 [1.83, 3.48], z = 5.614 %, p < 0.0001) and OS (HR = 1.97 [1.50, 2.58], z = 4.878, p < 0.0001). DTCs from BM were detected and correlated with PFS/DFS and OS in 3 studies [22, 34, 37]. The results showed that DTCs were also significantly associated with PFS/DFS (n = 2, HR = 1.60 [1.17, 2.19], z = 2.926, p < 0.0034) and OS (n = 2, HR = 1.89 [1.33, 2.68], z = 2.358, p < 0.0004). These suggested both CTCs and DTCs could be useful for evaluating prognostic value of ovarian cancer.

**Table 3:** Subgroup analyses of CTCs/DTCs

Subgroup analysis	PFS/DFS						OS					
	No. of Studies (sample size)	HR (95 % CI)	Model	*P* value	P-H	Begg’s test, p value	No. of Studies (sample size)	HR (95 % CI)	Model	*P* value	P-H	Begg’s test, p value
Total (CTCs and DTCs)	6 (885)	1.99 [1.59, 2.50]	FE	<.0001	0.1047	0.0167	7 (965)	1.94 [1.56 2.40]	FE	<.0001	0.7704	0.2389
Sampling from BM (DTCs)	2 (181)	1.60 [1.17, 2.19]	FE	0.0034	0.4417	1.000	2 (181)	1.89 [1.33, 2.68]	FE	0.0004	0.9687	1.0000
Sampling from PB (CTCs)	4 (704)	2.52 [1.83, 3.48]	FE	<.0001	0.2064	0.3333	5 (784)	1.97 [1.50, 2.58]	FE	<.0001	0.5147	0.0833
Total (IHC and RT-PCR)	5 (756)	1.91 [1.51, 2.40]	FE	<.0001	0.2037	0.0833	6 (818)	1.89 [1.52, 2.36]	FE	<.0001	0.7839	0.7194
Detected DTCs by IHC	3 (397)	1.70 [1.33, 2.19]	FE	<.0001	0.5934	1.0000	3 (397)	1.96 [1.48, 2.60]	FE	0.0004	0.9314	1.0000
Detected CTCs by RT-PCR	2 (359)	3.45 [1.95, 6.24]	FE	<.0001	0.9452	1.0000	3 (439)	1.78 [1.24, 2.54]	FE	0.0017	0.3463	0.3333

*FE* Fixed-Effects; *P-H* P-Heterogeneity

Four studies detected CTC by RT-PCR methods [17, 19, 26, 35]. Eleven studies detected by CellSearch system or other IHC methods [5, 6, 20–25, 34, 37, 38]. We divided the studies into molecular-based and immunological-based detection methods for sub-group analysis. For molecular-based subgroup, HR and 95 % CI for OS was 1.78 [1.24, 2.54] (z = 3.1369, p < 0.0001) among three studies [35, 19, 17] whereas HR and 95 % CI for PFS/DFS was 3.49 [1.95, 6.24] (z = 4.2007, p < 0.0001) among two studies [35, 19]. For immunological-based subgroup, HR and 95 % CI for OS and PFS/DFS was 1.96 [1.48, 2.60] (z = 3.5380, p < 0.0001) and 1.70 [1.33, 2.19] (z = 4.1628, p < 0.0001) amongst three studies [37, 34, 22], respectively. The results indicated that both RT-PCR and IHC detection methods were able to detect CTCs/DTCs in predicting patient’s survival.

**Fig. 4:**
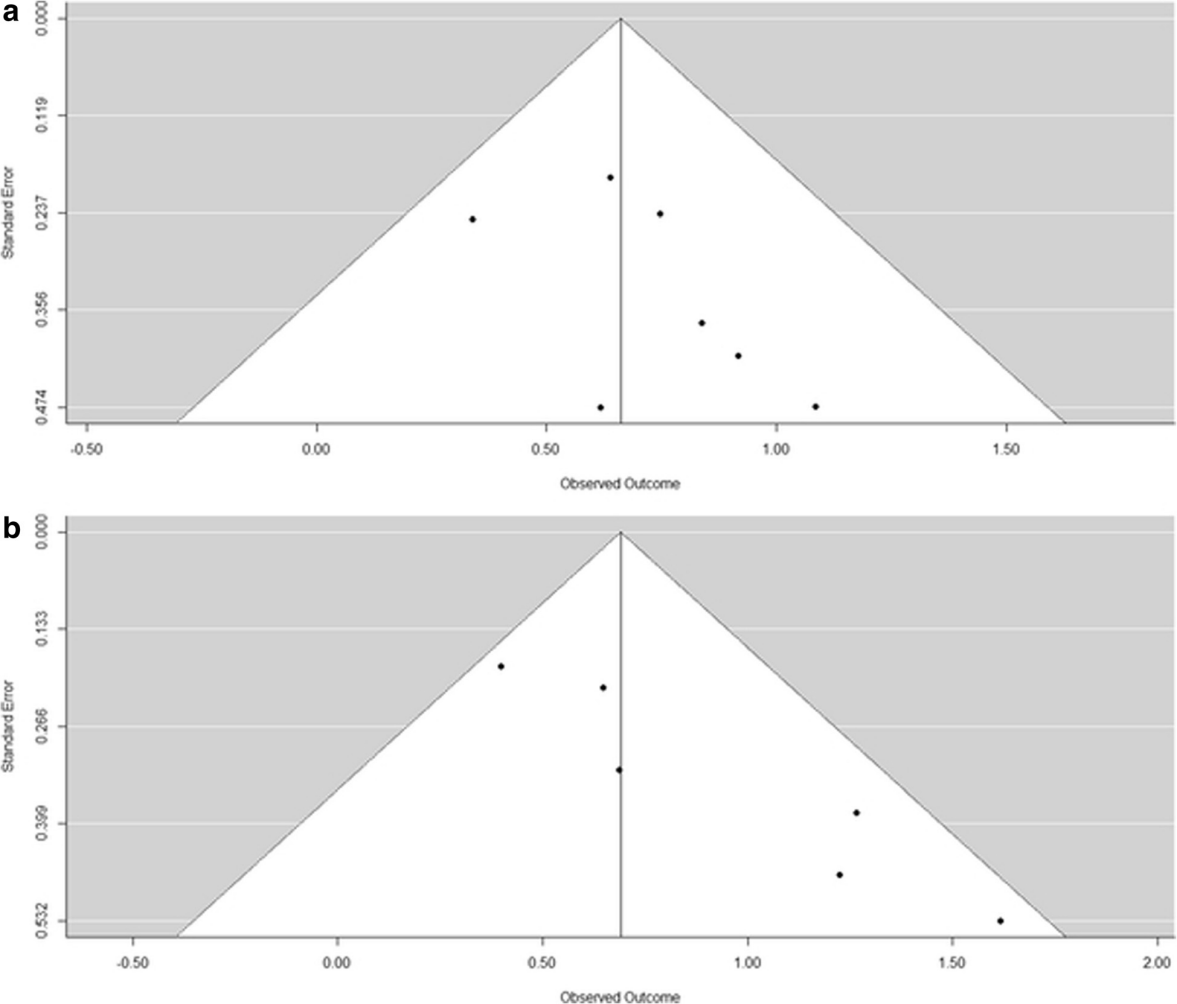
Begg’s funnel plots of publication bias summary. **a** Overall survival (OS); **b** Progression-free Disease-free survival (PFS/DFS)

Begg’s funnel plot was used to identify individual studies in relation to their respective standard deviation, which revealed no evidence of asymmetry (p = 0.2389) in an overall analysis of the studies of OS (Fig. 4a). There was no evidence of asymmetry (p = 0.0167) in an overall analysis of the studies of PFS/DFS (Fig. 4b). Using Egger’s test, there was no significant publication bias for overall analysis of the studies of OS (p = 0.3405), but significant bias was found for overall analysis of the studies of PFS/DFS (p = 0.0051). There was no publication bias for all subsequent subgroup analyses.

## Discussion

In our systematic review and meta-analysis, the results showed strong association of CTCs/DTCs not only with advanced staging and poor prognosis in patients with ovarian cancer, but also with treatment response. Although there was publication bias in Pearl et al. 2014 and Kuhlmann et al. 2014 studies for PFS/DFS group analysis, CTCs/DTCs were still significantly associated with PFS/DFS after bias removed. The combined HRs of CTCs/DTCs for OS and PFS/DFS was nearly 2.0, suggesting that the detected CTCs/DTCs had a strong predictive values for OS and PFS/DFS. For CTCs alone, the pooled HRs of CTCs for PFS/DFS were more than 2.0, indicating that CTCs could be used as a prognostic marker for ovarian cancer.

Association of CTCs/DTCs with clinopathological characteristics revealed that CTCs/DTCs significantly associated with advanced tumor stage and treatment response. No significant association were observed with histological subtypes, debulking surgery and lymph nodes metastasis. Experimental studies had demonstrated detection of CTCs were significantly correlated with the advanced stage [39, 40]. It may be one of the reasons why these patients have a high incidence of tumor recurrence after surgical resection. Residual disease after cyto-reduction surgery was associated with poor prognosis of ovarian cancer [41]. Moreover, in terms of the response to chemotherapy, CTCs/DTCs were significantly associated with treatment response. It suggested that CTCs/DTCs could be used as an early predictive marker of tumor response in ovarian cancer patients undergo chemotherapy. In our meta-analysis, detection of CTC was not significantly associated with the evidence of optimal or suboptimal surgery, only one study [35] reached a conclusion of positive (OR 2.31 [1.19-4.50]) with residual diseases by detecting DTC. This confusing result could be only resolved when more studies were conducted to confirm clinical values of the CTCs in ovarian cancer. On the other hand, ovarian cancer grows and recurs mainly in direct dissemination in the abdominal cavity [42]. Lymph nodes metastasis occur only when cancer cells invade lymphatic vessels while CTCs/DTCs occur only when cancer cells invade blood vessels. Although both lymph nodes metastasis and CTCs/DTCs were associated with poor prognosis in ovarian cancer patients [43], CTCs/DTCs were not significantly associated with lymph nodes metastasis, indicating the cancer cells may spread differently.

In subgroup analysis, comparing the HR of CTCs with the HR of DTCs for survival, CTCs for PFS/DFS was larger than DTCs for PFS/DFS (HR 2.50 vs 1.60) while CTCs for OS was similar to DTCs for OS (HR 1.97 vs 1.89). This indicated that CTCs could be more sensitive than DTC in evaluating tumor progression. In addition, detection of CTC seems more practical than DTC in terms of monitoring of progression of disease. Although bone marrow is the major site of metastasis [44], CTC could be systematically evaluated in peripheral blood stream. For CTCs/DTCs detection methods, IHC and RT-PCR were two main methods. Compared with IHC, although both methods were significantly associated with poor prognosis, RT-PCR seems to be more sensitive than IHC (HR 3.49 vs 1.70) [45]. This suggested that RT-PCR could be a promising methods in identifying CTC/DTC in patients with ovarian cancer. However, significant challenges include the frequency of both false positive and false negative results and the difficulty in quantitating relative levels of expression. Although genomic analyses of cell-free DNA fragments in peripheral blood have been reported [46, 47] and recently extended to the whole-genome scale [48], in situ and morphological analyses by fluorescent in situ hybridization (FISH) and IHC will be not possible. Apart from nucleic acid-based detection of CTC, detection and isolation of CTC by virtue of their physical properties distinguished from normal blood cells, including cell size, cell density, membrane charge and migratory properties, has advantageous to analyze CTCs with intact functional cancer cells circulating in peripheral blood [49]. It shows promising in CTC detection but requires further validation [50, 51].

CTCs/DTCs were associated with a poor survival outcome in our meta-analysis. However, there were limitations in this meta-analysis. Firstly, the number of patients in each study were relatively small. The results should be confirmed by larger prospectively clinical study. Secondly, the methodology varied in different studies lead to heterogeneity in experimental design, detection methods and defining the presence of CTCs/DTCs. However, there is still no gold standard in the definition of positive results in detection of CTCs/DTCs. And validation studies are still lacking. An international agreement of the definition of ‘positive’ CTCs in future trial is necessary. Thirdly, logHR and SE results were extracted from either multivariate analysis or univariate analysis studies. To ensure data integrity, we combined these univariate analysis and multivariate analysis together.

## Conclusion

In conclusion, available evidence supports that CTCs/DTCs were significantly associated with advanced tumor stage, residual diseases, and treatment response, but not with histological types and lymph node metastasis in patients with ovarian cancer. Moreover, CTCs also were significantly associated with a poorer survival. CTCs/DTCs could be a reliable non-invasive prognostic marker for ovarian cancer. Clinical management based on CTCs/DTCs could be useful for determining which patients would potentially benefit from adjuvant therapy.

## Additional files

Additional file 1:
**Search strategies and results of Embase.**
Additional file 2:
**Meta-analysis of Observational Studies in Epidemiology (MOOSE) Checklist.**
Additional file 3:
**Quality assessment of included cohort studies with the Newcastle-Ottawa Scale (NOS).**

## Abbreviations

CTCs: Circulating tumor cells
DTCs: Disseminated tumor cells
RT-PCR: Reverse transcription-polymerase chain reaction
IHC: Immunocytochemistry
MAGE-A: Melanoma-associated antigens A
PPIC: Peptidylprolyl isomerase C (cyclophilin C)
GPX8: Glutathione peroxidase 8
CDH3: Cadherin-3
TUSC3: Tumor suppressor candidate 3
COL3A1: Collagen, type III, alpha 1
LAMB1: Laminin subunit beta-1
MAM: Mammaglobin A
ESRP2: Epithelial splicing regulatory protein 2
AGR2: Anterior gradient protein 2 homolog
BAIAP2L1: Brain-specific angiogenesis inhibitor 1-associated protein 2-like protein 1
TFF1: Trefoil factor 1
EpCAM: Epithelial cell adhesion molecule
MUC-1: Mucin 1
MUC-16: Mucin 16
HER2: Human growth factor receptor 2
A45-B/B3: Pan-cytokeratin antibody (CK 8, 18, 19)
MOC-31: Epithelial glycoprotein 2 mouse monoclonal antibody
EGFR: Epithelial growth factor receptor
iCTC: Invasive circulating tumor cells
CK: Cytokeratin
CAM: Cell adhesion matrix
FISH: Fluorescent in situ hybridization
BM: Bone marrow
HR: Hazards ratio
OR: Odds ratio
lnHR: Natural logarithm of HR
SE: Standard error
CIs: Confidence intervals
OS: Overall survival
PFS/DFS: Progression-free survival/disease-free survival
