# Supplementary material for: Prognostic value of circulating tumor cells and disseminated tumor cells in patients with ovarian cancer: a systematic review and meta-analysis
Source: J Ovarian Res. 2015 Jun 16;8:38. doi: 10.1186/s13048-015-0168-9 (PMC4479068; doi:10.1186/s13048-015-0168-9)
Supplement: Additional file 1: — Search strategies and results of Embase. [file 13048_2015_168_MOESM1_ESM.doc]

**Additional file 1 –Search strategies and results of Embase**

1. Data base: **Embase Classic+Embase** (via OVIDSP platform)
2. Time span: < 1947 to 2015 April 27 >

Searches were performed on 2015-04-27 at the Library of The Chinese University of Hong Kong, Hong Kong

Search strategies and results

| **Set** | **Searches** | **Results** |
| --- | --- | --- |
| 1 | ((Blood or hemato* or heamato*) adj3 (tumo* cell* or cancer* cell* or carcinom* cell* or neoplas* cell*)).mp. [mp=title, abstract, subject headings, heading word, drug trade name, original title, device manufacturer, drug manufacturer, device trade name, keyword] | 18471 |
| 2 | ((Circulat* or isolated or disseminat* or occult or metastatic) adj3 (tumo* cell* or cancer* cell* or carcinom* cell* or neoplas* cell*)).mp. [mp=title, abstract, subject headings, heading word, drug trade name, original title, device manufacturer, drug manufacturer, device trade name, keyword] | 19669 |
| 3 | 2 OR 1 | 35527 |
| 4 | ((Ovar* or ovary* or ovaries*) adj3 (tumo* or cancer* or carcinom* or neoplas*)).mp. [mp=title, abstract, subject headings, heading word, drug trade name, original title, device manufacturer, drug manufacturer, device trade name, keyword] | 132366 |
| 5 | exp ovarian cancer/ | 84563 |
| 6 | 5 OR 4 | 135607 |
| 7 | 3 AND 6 | 1440 |
| 8 | (survival* or prognos* or recurren*).mp. [mp=title, abstract, subject headings, heading word, drug trade name, original title, device manufacturer, drug manufacturer, device trade name, keyword] | 2144438 |
| 9 | ((predict* or risk* or clinic*) adj3 (factor* or marker* or biomarker* or value* or role* or significan*)).mp. [mp=title, abstract, subject headings, heading word, drug trade name, original title, device manufacturer, drug manufacturer, device trade name, keyword] | 1427609 |
| 10 | exp prognosis/ | 504577 |
| 11 | 8 OR 9 OR 10 | 3292273 |
| 12 | 7 AND 11 | 636 |
| 13 | ((Ovar* or ovary* or ovaries*) adj3 (tumo* or cancer* or carcinom* or neoplas*)).ti. | 51402 |
| 14 | 12 AND 13 | 246 |
| 15 | (mouse or mice or rat* or animal*).ti. | 1890845 |
| 16 | 14 NOT 15 | 236 |

**Note.** mp: the term should appears in title, abstract, subject headings, heading word, drug trade name, original title, device manufacturer, drug manufacturer, device trade name, or keyword of the manuscript.

adj3: position operator, the term should be adjacent to the other with less than three words between them.

*: right-hand truncation

exp: explode

/: mesh term

ti: search terms in the title of the manuscript.
