# Supplementary material for: Prognostic value of circulating tumor cells and disseminated tumor cells in patients with ovarian cancer: a systematic review and meta-analysis
Source: J Ovarian Res. 2015 Jun 16;8:38. doi: 10.1186/s13048-015-0168-9 (PMC4479068; doi:10.1186/s13048-015-0168-9)
Supplement: Additional file 2: — Meta-analysis of Observational Studies in Epidemiology (MOOSE) Checklist. [file 13048_2015_168_MOESM2_ESM.doc]

**Meta-analysis of Observational Studies in Epidemiology (MOOSE) Checklist**

***Manuscript title: Prognostic Value of Circulating Tumor Cells and Disseminated tumor cells in Patients with Ovarian Cancer: A Systematic Review and Meta-analysis***

| **Criteria** | | **Brief description of how the criteria were handled in the meta-analysis** |
| --- | --- | --- |
| **Reporting of background should include** | |  |
| √ | Problem definition | The prognostic role of CTCs/DTCs remain controversial in ovarian cancer patients. |
| √ | Hypothesis statement | We hypothesize that CTCs/DTCs can predict the survival of patients with ovarian cancer. |
| √ | Description of study outcomes | Any prognostic outcomes that can be treated as overall survival (OS), progression-free survival/disease-free survival (PFS/DFS). |
| √ | Type of exposure or intervention used | Patients at exposure refer to those with detectable CTCs/DTCs or altered levels of molecular derivatives, which are identified by any kind of cytological and molecular methods. |
| √ | Type of study designs used | No limitations on study designs except non-research articles such as letters, editorial and short survey et al. |
| √ | Study population | No restrictions. |
| **Reporting of search strategy should include** | |  |
| √ | Qualifications of searchers | The credentials of the three investigators (who contributed to the search strategy) are indicated in the author list. |
| √ | Search strategy, including time period included in the synthesis and keywords | Embase Classic+Embase from 1947 to 2015 February 11  Medline from 1945 to 2015 February 11  See Additional file 1 |
| √ | Databases and registries searched | Embase Classic+Embase and Medline |
| √ | Search software used, name and version, including special features | OvidSP |
| √ | Use of hand searching | References of the retrieved papers were hand searched for additional studies. |
| √ | List of citations located and those excluded, including justifications | Details of the literature search process are outlined in the flow chart (Figure 1). The citation list of excluded articles is available upon request. |
| √ | Method of addressing articles published in languages other than English | We set no search restrictions on language and we obtained all included articles in English language. |
| √ | Method of handling abstracts and unpublished studies | We did not include unpublished or abstract only studies. |
| √ | Description of any contact with authors | We will contacted the original authors for clarification if necessary. |
| **Reporting of methods should include** | |  |
| √ | Description of relevance or appropriateness of studies assembled for assessing the hypothesis to be tested | Detailed inclusion and exclusion criteria have been described in the manuscript. |
| √ | Rationale for the selection and coding of data | Data extracted from each eligible study were any essential clinical factors, characteristics and survival data, which were relevant to the survival of ovarian cancer patients. |
| √ | Assessment of confounding | We mainly conducted Begg’s funnel plot to evaluate the confounding factors. See figure 4 |
| √ | Assessment of study quality, including blinding of quality assessors; stratification or regression on possible predictors of study results | We used the Newcastle Ottawa Scale (NOS) to assess the quality of each study. We conducted sensitivity analysis by removing the studies in low quality. See Additional file 3 |
| √ | Assessment of heterogeneity | We applied the Q statistic and p value to assess the heterogeneity. |
| √ | Description of statistical methods in sufficient detail to be replicated | We explained detailed methods and the software we used to process the data in the manuscript. |
| √ | Provision of appropriate tables and graphics | We included one flow chart to show the method of studies selection, figure 2-3 to show the forest plots, table 1 table to show the characteristics of included studies, table 2 to show the results of odds ratio of clinical and pathological variables, table 3 to show the results of subgroup analyses |
| **Reporting of results should include** | |  |
| √ | Graph summarizing individual study estimates and overall estimate | Figure 2-3 |
| √ | Table giving descriptive information for each study included | Table 1 |
| √ | Results of sensitivity testing | These were described in the manuscript and figure 4 |
| √ | Indication of statistical uncertainty of findings | 95% CI intervals were presented for all meta-analyses together with p values |
| **Reporting of discussion should include** | |  |
| √ | Quantitative assessment of bias | Begg and Egger’s test |
| √ | Justification for exclusion | Reasons for exclusion were shown in figure 1. |
| √ | Assessment of quality of included studies | See above mentioned parts. |
| **Reporting of conclusions should include** | |  |
| √ | Consideration of alternative explanations for observed results | We discussed in the manuscripts that heterogeneity existed in variability in definitions CTCs/DTCs presence, measurements, and experimental design might contribute to bias of included studies. |
| √ | Generalization of the conclusions | Our results evidenced the prognostic role of CTCs/DTCs in ovarian cancer. |
| √ | Guidelines for future research | Large prospective studies are needed to validate the prognostic values of CTCs/DTCs with multiple time points in homogeneous ovarian cancer patients. But above all, standardized detection platforms are expected to normalize and reduce the inconsistencies across studies. |
| √ | Disclosure of funding source | Funding sources were stated in the manuscript. |


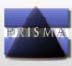
**PRISMA 2009 Checklist**

| **Section/topic** | **#** | **Checklist item** | **Reported on** |
| --- | --- | --- | --- |
| **TITLE** | | |  |
| Title | 1 | Identify the report as a systematic review, meta-analysis, or both. | Title |
| **ABSTRACT** | | |  |
| Structured summary | 2 | Provide a structured summary including, as applicable: background; objectives; data sources; study eligibility criteria, participants, and interventions; study appraisal and synthesis methods; results; limitations; conclusions and implications of key findings; systematic review registration number. | Abstract |
| **INTRODUCTION** | | |  |
| Rationale | 3 | Describe the rationale for the review in the context of what is already known. | Introduction |
| Objectives | 4 | Provide an explicit statement of questions being addressed with reference to participants, interventions, comparisons, outcomes, and study design (PICOS). | Introduction |
| **METHODS** | | |  |
| Protocol and registration | 5 | Indicate if a review protocol exists, if and where it can be accessed (e.g., Web address), and, if available, provide registration information including registration number. | N/A |
| Eligibility criteria | 6 | Specify study characteristics (e.g., PICOS, length of follow-up) and report characteristics (e.g., years considered, language, publication status) used as criteria for eligibility, giving rationale. | Materials and Methods |
| Information sources | 7 | Describe all information sources (e.g., databases with dates of coverage, contact with study authors to identify additional studies) in the search and date last searched. | Materials and Methods |
| Search | 8 | Present full electronic search strategy for at least one database, including any limits used, such that it could be repeated. | Materials and Methods |
| Study selection | 9 | State the process for selecting studies (i.e., screening, eligibility, included in systematic review, and, if applicable, included in the meta-analysis). | Materials and Methods |
| Data collection process | 10 | Describe method of data extraction from reports (e.g., piloted forms, independently, in duplicate) and any processes for obtaining and confirming data from investigators. | Materials and Methods |
| Data items | 11 | List and define all variables for which data were sought (e.g., PICOS, funding sources) and any assumptions and simplifications made. | N/A |
| Risk of bias in individual studies | 12 | Describe methods used for assessing risk of bias of individual studies (including specification of whether this was done at the study or outcome level), and how this information is to be used in any data synthesis. | Materials and Methods |
| Summary measures | 13 | State the principal summary measures (e.g., risk ratio, difference in means). | Materials and Methods |
| Synthesis of results | 14 | Describe the methods of handling data and combining results of studies, if done, including measures of consistency (e.g., I^2^) for each meta-analysis. | Materials and Methods |

Page 1 of 2

| **Section/topic** | **#** | **Checklist item** | **Reported on page #** |
| --- | --- | --- | --- |
| Risk of bias across studies | 15 | Specify any assessment of risk of bias that may affect the cumulative evidence (e.g., publication bias, selective reporting within studies). | Materials and Methods |
| Additional analyses | 16 | Describe methods of additional analyses (e.g., sensitivity or subgroup analyses, meta-regression), if done, indicating which were pre-specified. | Materials and Methods |
| **RESULTS** | | |  |
| Study selection | 17 | Give numbers of studies screened, assessed for eligibility, and included in the review, with reasons for exclusions at each stage, ideally with a flow diagram. | Results & Figure 1 |
| Study characteristics | 18 | For each study, present characteristics for which data were extracted (e.g., study size, PICOS, follow-up period) and provide the citations. | Table 1 |
| Risk of bias within studies | 19 | Present data on risk of bias of each study and, if available, any outcome level assessment (see item 12). | Results & Figure 4 |
| Results of individual studies | 20 | For all outcomes considered (benefits or harms), present, for each study: (a) simple summary data for each intervention group (b) effect estimates and confidence intervals, ideally with a forest plot. | Results & Figure 2,3 |
| Synthesis of results | 21 | Present results of each meta-analysis done, including confidence intervals and measures of consistency. | Results |
| Risk of bias across studies | 22 | Present results of any assessment of risk of bias across studies (see Item 15). | Results |
| Additional analysis | 23 | Give results of additional analyses, if done (e.g., sensitivity or subgroup analyses, meta-regression [see Item 16]). | Results & Table 2,3 |
| **DISCUSSION** | | |  |
| Summary of evidence | 24 | Summarize the main findings including the strength of evidence for each main outcome; consider their relevance to key groups (e.g., healthcare providers, users, and policy makers). | Discussion |
| Limitations | 25 | Discuss limitations at study and outcome level (e.g., risk of bias), and at review-level (e.g., incomplete retrieval of identified research, reporting bias). | Discussion |
| Conclusions | 26 | Provide a general interpretation of the results in the context of other evidence, and implications for future research. | Discussion |
| **FUNDING** | | |  |
| Funding | 27 | Describe sources of funding for the systematic review and other support (e.g., supply of data); role of funders for the systematic review. | Acknowledgements |

*From:*  Moher D, Liberati A, Tetzlaff J, Altman DG, The PRISMA Group (2009). Preferred Reporting Items for Systematic Reviews and Meta-Analyses: The PRISMA Statement. PLoS Med 6(6): e1000097. doi:10.1371/journal.pmed1000097

For more information, visit: **www.prisma-statement.org**.

Page 2 of 2
