# Supplementary material for: Prognostic value of circulating tumor cells and disseminated tumor cells in patients with ovarian cancer: a systematic review and meta-analysis
Source: J Ovarian Res. 2015 Jun 16;8:38. doi: 10.1186/s13048-015-0168-9 (PMC4479068; doi:10.1186/s13048-015-0168-9)
Supplement: Additional file 3: — Quality assessment of included cohort studies with the Newcastle-Ottawa Scale (NOS). [file 13048_2015_168_MOESM3_ESM.doc]

Additional file 3– Quality assessment of included cohort studies with the Newcastle-Ottawa Scale (NOS)

| **Studies** | **Score for Selection** | | | | **Score for Comparability** | | **Score for Outcome** | | | **Aggregate score** | **Quality** |
| --- | --- | --- | --- | --- | --- | --- | --- | --- | --- | --- | --- |
|  | **Item 1** | **Item 2** | **Item 3** | **Item 4** | **Item 1** | **Item 2** | **Item 1** | **Item 2** | **Item 3** |  |  |
| (Sang, Wu et al. 2014) | 1 | 1 | 1 | 1 | 0 | 0 | 1 | 0 | 1 | 6 | High |
| (Pearl, Zhao et al. 2014) | 1 | 1 | 1 | 1 | 0 | 0 | 1 | 1 | 1 | 7 | High |
| (Kuhlmann, et al. 2014) | 1 | 1 | 1 | 1 | 0 | 0 | 1 | 0 | 1 | 6 | High |
| (Obermayr, et al. 2013) | 1 | 1 | 1 | 1 | 0 | 0 | 1 | 0 | 0 | 5 | High |
| (Poveda, et al. 2011) | 1 | 1 | 1 | 1 | 0 | 0 | 1 | 0 | 1 | 6 | High |
| (Aktas, et al. 2011) | 1 | 1 | 1 | 1 | 1 | 0 | 0 | 0 | 0 | 5 | High |
| (Schindlbeck, et al. 2007) | 1 | 1 | 1 | 1 | 0 | 0 | 1 | 0 | 0 | 5 | High |
| (Banys, et al. 2009) | 1 | 1 | 1 | 1 | 0 | 0 | 1 | 0 | 1 | 6 | High |
| (Wimberger, et al. 2011) | 1 | 1 | 1 | 1 | 0 | 0 | 1 | 0 | 0 | 5 | High |
| (Fehm, et al. 2013) | 1 | 1 | 1 | 1 | 0 | 0 | 1 | 0 | 0 | 5 | High |
| (Marth, et al. 2002) | 1 | 1 | 1 | 1 | 0 | 0 | 1 | 0 | 0 | 5 | High |
| (Judson, et al. 2003) | 1 | 1 | 1 | 1 | 0 | 0 | 1 | 0 | 1 | 6 | High |
| (Fan, et al. 2009) | 1 | 1 | 1 | 1 | 0 | 0 | 1 | 1 | 0 | 6 | High |
| (Behbakht, et al. 2011) | 1 | 1 | 1 | 1 | 0 | 0 | 1 | 0 | 0 | 5 | High |
| (Wimberger, et al. 2007) | 1 | 1 | 1 | 1 | 0 | 0 | 1 | 0 | 1 | 6 | High |
| (Fehm, et al. 2006) | 1 | 1 | 1 | 1 | 0 | 0 | 1 | 1 | 0 | 6 | High |
| **Note.** Numbered items in each category of the NOS are listed below. | | | | | | | | | | | |

**Selection**

Item 1) Representativeness of the exposed cohort

Item 2) Selection of the non-exposed cohort

Item 3) Ascertainment of exposure

Item 4) Demonstration that outcome of interest was not present at start of study

**Comparability**

Comparability of cohorts on the basis of the design or analysis

Item 1) study controls for the most important factor (i.e., age)

Item 2) study controls for any additional factor (treatments for cancer)

**Outcome**

Item 1) Assessment of outcome

Item 2) Was follow-up long enough for outcomes to occur (maximum follow-up period was over 36 month)

Item 3) Adequacy of follow up of cohorts (over 90%)

**Reference**

Wells G, Shea B, O’connell D, Peterson J, Welch V, Losos M, Tugwell P: **The Newcastle-Ottawa Scale (NOS) for assessing the quality of nonrandomised studies in meta-analyses**. In*.*; 2000.
